# Root Cause Analysis of Degradation in Protonic Ceramic Electrochemical Cell with Interfacial Electrical Sensors Using Data‐Driven Machine Learning

**DOI:** 10.1002/advs.202304074

**Published:** 2023-08-26

**Authors:** Wei Wu, Congjian Wang, Wenjuan Bian, Bin Hua, Joshua Y. Gomez, Christopher J. Orme, Wei Tang, Frederick F. Stewart, Dong Ding

**Affiliations:** ^1^ Energy & Environmental Science and Technology Idaho National Laboratory Idaho Falls ID 83415 USA; ^2^ Nuclear Science and Technology Idaho National Laboratory Idaho Falls ID 83415 USA

**Keywords:** degradation prediction, machine learning, protonic ceramic electrochemical cell, root cause analysis, steam electrolysis

## Abstract

Protonic ceramic electrochemical cells (PCECs) offer promising paths for energy storage and conversion. Despite considerable achievements made, PCECs still face challenges such as physiochemical compatibility between componenets and suboptimal solid–solid contact at the interfaces between the electrolytes and electrodes. In this study, a novel approach is proposed that combines in situ electrochemical characterization of interfacial electrical sensor embedded PCECs and machine learning to quantify the contributions of different cell components to total degradation, as well as to predict the remaining useful life. The experimental results suggest that the overpotential induced by the oxygen electrode is 48% less than that of oxygen electrode/electrolyte interfacial contact for up to 1171 h. The data‐driven machine learning simulation predicts the RUL of up to 2132 h. The root cause of degradation is overpotential increase induced by oxygen electrode, which accounts for 82.9% of total cell degradation. The success of the failure diagnostic model is demonstrated by its consistency with degradation modes that do not manifest in electrolysis fade during early real operations. This synergistic approach provides valuable insights into practical failure diagnosis of PCECs and has the potential to revolutionize their development by enabling improved performance prediction and material selection for enhanced durability and efficiency.

## Graphical abstract

A method that combines electrochemical analysis and machine learning is proposed to accurately identify degradation sources and predict the remaining life of protonic ceramic electrochemical cells (PCEC) during steam electrolysis. This approach uncovers degradation mechanisms, and aids in mitigation strategy development for commercializing PCEC technology. It also demonstrates the potential of data‐driven machine learning in understanding complex solid‐state systems.

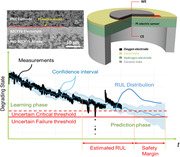

## 1 Introduction

The solid oxide cells (SOC) play a vital role in the energy‐to‐molecules/materials pillar by utilizing energy associated with renewables and nuclear power to produce functional intermediates, fuels, and chemicals.^[^ ^1^ ^]^ Among which, protonic ceramic electrochemical cells (PCECs) have attracted a significant amount of interest from research communities for several decades due to their lower operating temperature compared with oxygen‐ion conducting ceramic electrochemical cells.^[^ ^2^ ^]^ The reduced operating temperatures mitigate many problems such as slow start‐up, fast degradation of cell/stack performance, the burden of design, and the cost of thermal insulation required by high temperatures.^[^ ^3^ ^]^ Like its counterpart oxygen‐ion conducting electrochemical cell, one of the major hurdles for PCEC commercialization is the longtime stability and the difficulty in real‐time characterizing and predicting failure mechanisms since they are operated in harsh environments including, but not limited to, elevated temperatures, a variety of gas compositions (high steam, hydrocarbons, hydrogen, etc), as well as corresponding reactions.^[^ ^4^ ^]^ Main degradation mechanisms of oxygen‐ion conducting electrochemical cells include the oxygen electrode delamination and nickel migration and coarsening in hydrogen electrode side.^[^ ^5^ ^]^ For PCEC degradation, various mechanisms have been proposed, primarily based on current and voltage measurements over time or impedance spectroscopy measurements taken at different temperatures before and after durability testing.^[^ ^6^ ^]^ A PCEC is very sensitive to operating conditions like temperature and gaseous feedstock. The degradation mechanism of PCECs is complex, particularly in the case of high‐temperature steam electrolysis used to produce hydrogen, where different components exhibit different degradation mechanisms. Additionally, the rate of degradation is greatly influenced by factors such as temperature, steam concentration, and applied voltage. Addressing these challenges will require ongoing research and development focused on optimizing operating conditions and identifying novel materials and fabrication methods that enhance the longevity and stability of PCECs. Attributed to ongoing breakthroughs in materials design, structure engineering, and operation parameters optimization, the reliability of PCECs has been substantially improved.^[^ ^7^ ^]^ Additionally, Sullivan et al. demonstrated progress in improving the durability of proton‐conducting ceramic fuel cells over 2500 h by applying a GDC interlayer at the cathode/electrolyte interface.^[^ ^8^ ^]^ However, these improved durability levels also pose new challenges for accurate early prognostics of PCEC lifetime due to typically nonlinear degradation behavior and the relatively limited electrochemical datasets available. As such, continued research and development will be necessary to optimize prognostic models and identify new materials and fabrication methods that further enhance the longevity and stability of PCECs.

In recent years, prognostics have been successfully applied in various applications, including SOCs, to predict the remaining service life and risk for future failure modes.^[^ ^9^ ^]^ Prognostics also play a crucial role in the life and cost management of SOCs, as the improved durability brings new challenges for accurate early prognostics.^[^ ^10^ ^]^ Two successful approaches, model‐based and data‐based, have been applied in fuel cell systems.^[^ ^11^ ^]^ Model‐based approaches use mathematical equations to describe the degradation process of the system, requiring precise knowledge of the system failure mechanisms. Data‐based approaches use monitored historical data to describe the degradation process.^[^ ^12^ ^]^ Most research in the literature reports degradation prediction through building semi‐empirical models.^[^ ^13^ ^]^ However, semi‐empirical models depend on experimental measurements and must be adapted for different types of electrochemical cells to predict performance degradation accurately.

Machine learning methods have been used in many engineering aspects including fuel cell stack and system prognostics.^[^ ^10^, ^14^ ^]^ The potential advantage of using machine learning with multiple cells lies in the ability to generalize and capture broader trends and variations across different cells and deterioration mechanisms. When considering a single cell, machine learning can still yield valuable insights into degradation behavior and enable accurate prognostics. This is due to its ability to uncover hidden patterns and relationships that may not be readily discernible using conventional data analysis methods. Moreover, the multi‐component configuration of PCEC (i.e., functional electrodes and electrolyte) makes it challenging to identify the degradation of each component during operation. To improve the adaptability and accuracy of the degradation model, data‐driven degradation predictions and diagnostic methods with an internal degradation indicator reflecting intrinsic degradation levels in dynamic operating conditions are preferred.

**Figure 1:**
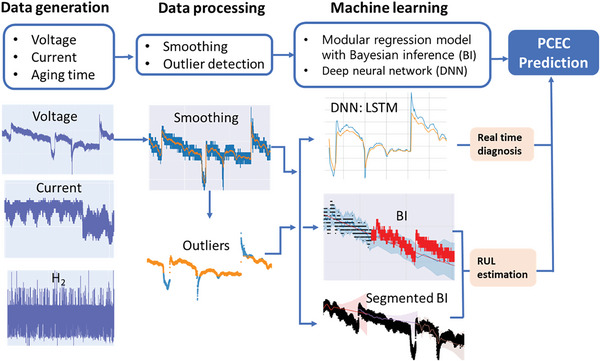
Machine learning processes for degradation prediction. Graphs are generated using data from ref. [20]. I. Sensor data collection (left); II. Data processing including smoothing and outlier detection (middle); III. Training and prediction through machine learning models, i.e., deep neural network, Modular regression model with Bayesian inference for uncertainty estimation, and segmented models when the signal is discontinuous (right).

Degradation in a SOC is often characterized by abnormal voltage, which may be higher (due to issues such as cell component delamination, isolating secondary phase formation, or coking) or lower/negative (due to electrolyte electronic leakage). These voltage abnormalities are typically the first signs of SOC failure.^[^ ^15^ ^]^ Three‐electrode configuration with the use of a reference electrode at a fixed potential has been an effective way to measure overpotential of a specific electrode in both electrochemical systems involving liquid phase electrolyte^[^ ^16^ ^]^ and oxygen‐ion conducting solid oxide fuel cell systems.^[^ ^17^ ^]^ However, application of such three‐electrode configuration to solid electrochemical cells requires extra caution. The modeling results suggest that the symmetry of the working and counter electrodes is the key factor determining if a reference electrode is suitable to use.^[^ ^18^ ^]^ Interfacial sensors are modified reference electrodes that are situated at specific electrode/electrolyte interfaces within asymmetric cell configurations, such as anode‐supported solid oxide electrochemical cells. In 2014, a method was developed to quantify the effects of different components on SOFC degradation by embedding micro‐electrical sensors into the interface between different components. This allowed for monitoring of current/voltage response and collection of impedance electrochemical data during operation.^[^ ^19^ ^]^ These micro‐electric sensors, embedded into the internal triple‐phase boundary (TPB), are crucial for measuring cell components individually and for quantifying their contributions to cell degradation. Furthermore, such sensors enable simultaneous failure diagnosis of SOCs based on advanced artificial intelligence (AI) technology (such as stacked sparse autoencoder, grid long short‐term memory, and physics‐informed neural networks). Considering the current development status of PCEC with no commercially scaled unit cell available, we transferred the experience in ref. 18 to 1″ button PCEC cell and reported a simple yet reliable data‐driven modeling method for analyzing the PCEC component degradation mechanism and predicting its lifetime during high‐temperature steam electrolysis for hydrogen production. The working concept is depicted in **Figure** **1**.

As shown in Figure 1, we have utilized machine learning algorithms, such as long short‐term memory (LSTM) networks, for early failure detection and long‐term degradation analysis in our PCEC system. The data was preprocessed by smoothing and removing outliers to enhance its quality. The modular regression model showed promising results for capturing long‐term degradations, as it addresses the changing degradation rates over time that can challenge traditional data analysis methods. By incorporating changepoints, the model measures degradation rate changes and selects the final growth rate for future predictions. Additionally, the modular regression model integrates well with Bayesian inference (BI) to predict trend uncertainties. In cases where calibrations affect degradation behaviors and cause discontinuous signals, we explored the segmented modular regression approach with BI. We emphasize that the machine learning models are trained on a comprehensive dataset that includes oxygen electrode, full‐cell, half‐cell, and interface. This enables the models to learn patterns and correlations between different components and their degradation behaviors. These datasets will be compared with current predictions from ML models to identify any anomaly behaviors or early failures. If no anomalies or signs of early failures are detected, the data will be included in the training dataset and utilized for future predictions. Additionally, offline re‐training can be conducted to enhance the forecast capabilities of the ML models.

## 2 Results and Discussion

### 2.1 PCEC Components Degradation Behavior

**Figure 2:**
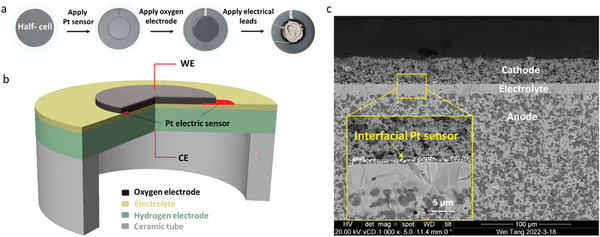
a) Digital photos of the cells with Pt sensor and reference electrode. b) Schematical illustration of the cell fabrication process. c) Cross‐sectional SEM image of full cell showing locations of electrical leads in full cell.

The morphology of the interfacial electric sensors at the cathode/electrolyte interface was examined using a field emission scanning electron microscope (SEM) (FEI QUENTA FEG 650). The deposition of interfacial electrical sensor was demonstrated in **Figure** **2**a. The test configuration with cross‐sectional image of as‐prepared cell was demonstrated in Figure 2b. Figure 2c presents the cross‐section of the fabricated unit cell, revealing a uniform distribution of the electric sensors with an average thickness of approximately 2.5 µm. The thickness of the electrolyte, PNC cathode, and Pt probe is 11, 27, and 2 µm, respectively. No interfacial delamination was observed between the electrodes and electrolyte, indicating good physical integrity of the designed cell.

**Figure 3:**
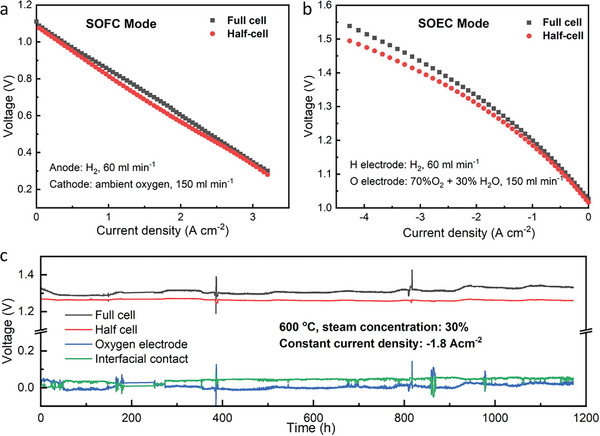
*I–V* curves of full‐cell and half‐cell in a) fuel‐cell and b) steam electrolysis mode. c) Long‐term stability of full‐cell, half‐cell, cathode, and interface under −1.8 A cm^−2^ at 600 °C with 30% steam.

**Figure** **3**a,b shows the electrochemical I‐V curves of both the full‐cell and half‐cell under both fuel cell and electrolysis modes at 600 °C. For fuel cell operation, hydrogen at a flow rate of 60 mL min^−1^ and ambient oxygen at a flow rate of 150 mL min^−1^ were fed into the anode and cathode, respectively. The cell exhibited a peak power density of 1.21 W cm^−2^ at 600 °C (Figure S1, Supporting Information), which is comparable to the values reported in our previous work.^[^ ^2b^ ^]^ This suggests that the physical disintegration induced by the Pt electric sensor has negligible effects on the performance of the cell. The open circuit voltage (OCV) of the full‐cell was measured to be 1.088 V, demonstrating good sealing effects and cell reproducibility. The OCV of the half‐cell was found to be 1.071 V, accounting for 98.4% of the total OCV. Figure 3b displays the *I–V* curves of both the full‐cell and half‐cell during electrolysis. The voltage variation between the full‐cell and half‐cell, denoted as ∆*U* = *U*_f –_ *U*_h_, stabilized at 0.01 V below current densities of −0.5 A cm^−2^. As the current density increased to −1.8 Acm^−2^, the voltages of *U*_f_ and *U*_h_ were measured to be 1.31 and 1.29 V, respectively. The corresponding overpotentials were 0.18 and 0.17 V for *U*_f_ and *U*_h_, respectively. The overpotential of the half‐cell at −1.8 A cm^−2^ accounted for 94.4% of the full‐cell. The exceptional initial activity of PNC during steam electrolysis is indicated by the limited overpotential induced by the PNC oxygen electrode and interfacial contact, resulting in outstanding oxygen reduction reaction (ORR) performance.

Generally, as described in Equation 1, the resistance of full‐cell (*R*_f_) includes the resistances of half‐cell (*R*_h_), oxygen electrode (*R*_o_), and interfacial contact (*R*_i_).

(1) $$\;R_{f}=R_{h}\;+R_{o}+R_{i}$$

The corresponding voltage can be denoted as:

(2) $$\;V_{f}=V_{h}\;+V_{o}+V_{i}$$

Therefore, the interfacial contact voltage, which reflects *R*_i_, can be determined by:

(3) $$\;V_{i}=V_{f}\;-V_{o}-V_{h}$$

We conducted long‐term electrolysis operation at 600 °C with a steam concentration of 30% (Figure 3c) at 1.331 V. During the initial 55‐hour operation, the full‐cell voltage decreased gradually to 1.291 V, which is a “break‐in” or “activation” phenomenon that has been also reported in PCEC research with 3D oxygen electrode.^[^ ^2d^ ^]^ The half‐cell voltage remained constant during the break‐in period, while the voltage of the oxygen electrode dropped from 0.036 to −0.013 V, confirming that the activation was associated with the oxygen electrode. Negative overpotential of oxygen electrode may result from electrochemical reaction kinetics: the kinetics of the electrochemical reaction at the triple‐phase boundary (TPB) of bulk oxygen electrode are faster than that at oxygen electrode/electrolyte interface.^[^ ^21^ ^]^ However, the impact of the introduced interfacial Pt electric sensor on the activation behavior of the oxygen electrode is unknown, despite its negligible effect on the overall cell performance.

**Figure 4:**
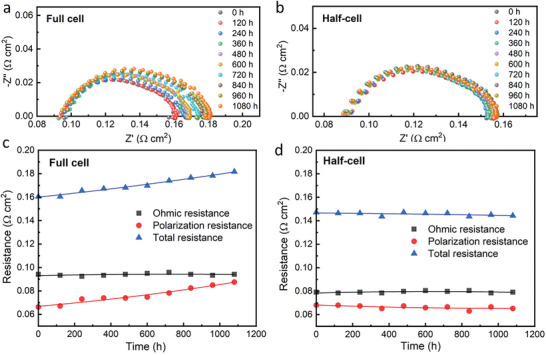
EIS results of a) full‐cell, b) half‐cell, and under OCV condition. And Resistance versus time of c) full‐cell, d) half‐cell.

In this research, we use voltages after “break‐in period” (≈55 h) as initial values. Therefore, the initial voltages of *V*_f_, *V*_h_, *V*_o_, and *V*_i_ were measured as 1.291,1.264, −0.013, and 0.040 V, respectively. The variation of *V*_f_, *V*_h_, *V*_o_, and *V*_i_ are listed in **Table** **1**. After 1171 h of operation, the degradation of V_f_ was estimated to be ≈3.17% (0.041 V), while the voltage of the half‐cell (*V*_h_) remained a negative degradation. Furthermore, the interfacial contact voltage *V*_i_ and oxygen electrode voltage Vo showed a degradation of 0.01 and 0.034 V, respectively. The voltage variation of oxygen electrode accounts for 82.9% of total voltage increase. These findings suggest that the primary source of degradation in the full‐cell is the PNC bulk oxygen electrode rather than PNC/electrolyte interface. **Figure** **4**a,b present the results of electrochemical impedance spectroscopy (EIS) for the full‐cell, half‐cell under OCV condition. The increase in polarization resistance of the full‐cell is evident, while the resistance of the half‐cell remained stable throughout the operation. Figure 4c,d shows the variation of their Ohmic resistance, polarization resistance, and total resistance over time. The Ohmic resistance of the cell remained unchanged, while the increase in polarization resistance contributed to the overall cell degradation. Considering that both the Ohmic and polarization resistance of the half‐cell showed limited variation, the increase in PNC polarization resistance is identified as the root cause for the total cell degradation.

**Table 1:** Variation of voltage of different components over time

Voltages	Initial voltage, [V]	Final voltage, [V]	Variation, ΔV	[% ]to total degradation
full‐cell, *V*_f_	1.291	1.332	0.041	100%
half‐cell, *V*_h_	1.264	1.261	−0.003	−7.3%
electrode, *V*_o_	−0.013	0.021	0.034	82.9%
interfacial, *V*_i_	0.040	0.050	0.010	2.4%

### 2.2 Degradation Prediction Using Machine Learning

To measure the accuracy in the model predictions, four different metrics, i.e., mean squared error (MSE), mean absolute error (MAE), r2 score (R2), and mean absolute percentage error (MAPE), are employed. Their mathematical definitions can be expressed as:

(4) $$MSE\;\left(V,\;\hat{V}\right)=\frac{1}{N}\;\sum_{i\;=\;0}^{N-1}{\left(V_{i}-{\hat{V}}_{i}\right)}^{2}$$

(5) $$MAE\;\left(V,\;\hat{V}\right)=\frac{1}{N}\;\sum_{i\;=\;0}^{N-1}\left|V_{i}-\hat{V_{i}}\right|$$

(6) $$R2\;\left(V,\;\hat{V}\right)=\;1-\sum_{i\;=\;1}^{N}{\left(V_{i}-{\hat{V}}_{i}\right)}^{2}/\sum_{i\;=\;1}^{N}{\left(V_{i}-{\bar{V}}_{i}\right)}^{2}$$

(7) $$MAPE\;\left(V,\;\hat{V}\right)=\frac{1}{N}\;\sum_{i\;=\;0}^{N-1}\left|V_{i}-{\hat{V}}_{i}\right|/\max\left(\varepsilon ,\;\left|V_{i}\right|\right)$$

where *V*_i_ is the measured data, ${\hat{V}}_{i}$ is the predicted value at time *t*_i_, *N* is the total number of time steps, and ε is a small positive number to avoid undefined results when *V* is zero.

Based on Figure 3c, it is observed that the voltages for the PCEC system did not stabilize initially, and there is some missing data between 200 and 300 h due to connection issues. Another observation from the full‐cell voltage data is that the voltage starts to slowly increase from 400 h, indicating that the PCEC system may have started to degrade. For the subsequent analysis, only the data from the degradation stage are used. Figure 3c also shows that the measured data contains large narrow spikes and dips, which can negatively impact the accuracy of long‐term predictions. To address this issue, robust covariance, and isolation forest techniques can be employed to automatically detect these abnormal behaviors, as demonstrated in Figure S2 (Supporting Information). In these plots, isolation forest is utilized to identify outliers, which are marked with blue dots. These outliers are then removed from the measured data, and a smoothing technique such as LOWESS is applied to the remaining data to eliminate noise and improve prediction accuracy. The smoothed data with a smoothing fraction of 0.1% is plotted with black lines, and these smoothed data points will be used for subsequent analysis in short‐term and long‐term predictions.

#### 2.2.1 Deep Neural Network Model for Short‐Term Prediction

**Figure 5:**
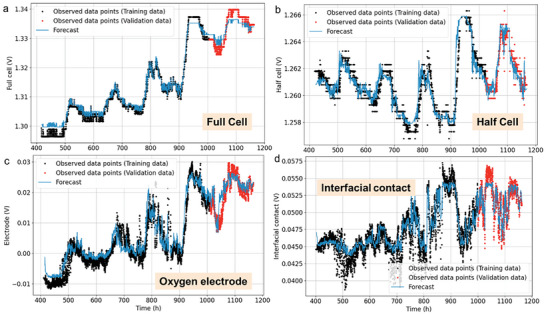
Voltage predictions for next one hour using LSTM.

The deep neural network model, i.e., LSTM model, is applied to the processed data at the degradation stage. The structure of the LSTM network is listed in Table S1 (Supporting Information), where the “dense layer” is a fully connected layer connecting each neuron to all neurons from the next layer, and the “lambda layer” is used to scale the outputs of the previous layer (i.e., the dense layer). The LSTM model was trained using the first 80% of the processed data, and the remaining 20% was used for validation to assess the prediction accuracy. The input window size for the data was set to 24 h, the forecast width was set to 1 h, and the shift was set to (1 h, 24 h), corresponding to 1‐hour and 1‐day predictions, respectively. The learning rate was set to 0.001, and the “adam” algorithm was used as the optimizer for the LSTM model. The predictions obtained from the LSTM model are illustrated in **Figure** **5** (1‐hour prediction) and Figure S3 (Supporting Information) (1‐day prediction), where the training data are highlighted with black dots, the validation data are highlighted with red dots, and the forecasts are plotted with a blue line. The accuracies of the predictions for the validation data are summarized in Table S2 (Supporting Information). The LSTM model performs accurately in the short‐term predictions for all four voltage predictions. Since the LSTM model demonstrates better performance in short‐term predictions, it can be used as a tool to detect anomaly behaviors in the PCEC system.

#### 2.2.2 Modular Regression Model for Long‐Term Prediction

**Figure 6:**
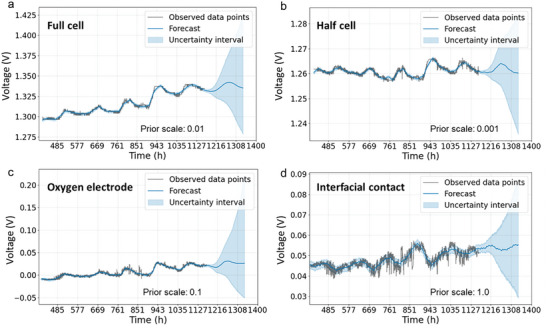
Voltage prediction with uncertainty using modular regression model.

In this section, the modular regression model (i.e., Prophet model) is used to predict the PCEC components voltages. The changepoints, which indicate points where the growth rates of the full‐cell voltage trend change, are automatically selected by the Prophet model. As depicted in **Figure** **6**, the growth rates of the full‐cell voltage trend change gradually over time. We assume that the final growth rate can be used for future extrapolation. To control the flexibility of the trend and the magnitude of changes at the changepoints, we employ a changepoint prior scale parameter. A smaller value of the changepoint prior scale indicates less variability in the trend changes, while a larger value indicates greater variability. In extreme cases, a large changepoint prior scale can result in capturing the seasonality as well. In order to avoid overfitting on a small number of final points, we set the final trend segment to 20% of the training data. Furthermore, we manually adjust the changepoint prior scale to ensure that the model accurately represents the data, taking into consideration the aforementioned metrics. The predictions for the voltages of the full‐cell, half‐cell, electrode, and interfacial contact are presented in Figure 6. These plots depict the models' predictions of short‐term degradations (i.e., 1 week after the last measurement) along with the variations on the trend predictions (i.e., ±*σ*, where σ represents the standard deviation). Additionally, Table S3 (Supporting Information) presents the accuracy of the models on the existing measurement data. As evident from both the table and the plots, the models effectively capture the trends and variations in the data, demonstrating their accuracy in predicting the voltages of the PCEC components.

**Figure 7:**
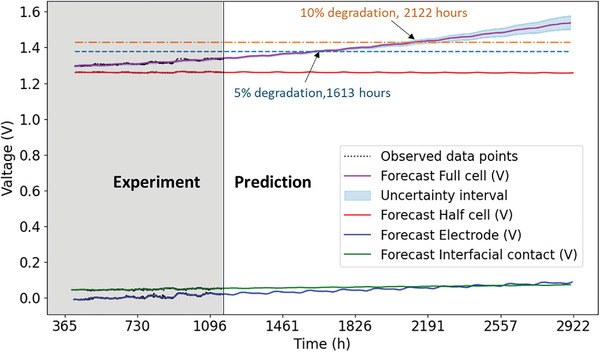
Data‐driven performance predictions for PCEC system.

The PCEC system is continuously monitored, and new degradation data is continuously collected. In such cases, the model can be retrained regularly with the updated degradation data to improve its performance. One observation from Figure 6 is that the uncertainties in the forecasts increase rapidly over time. To address this issue, the value of the changepoint prior scale can be reduced to narrow down the uncertainty bounds. As mentioned earlier, a smaller value of the changepoint prior scale indicates less flexibility in the trend estimation, and reducing its value will also decrease the variance on the trend. In other words, the trend will be underfitting to the local data, but it can capture the overall degradation behavior, making the model suitable for long‐term predictions (i.e., RUL estimation). We have demonstrated these arguments in **Figure** **7**, where only the changepoint prior scale value for the full cell is reduced from 0.01 to 0.001, while keeping the values for other predictions the same as reported in Figure 6. Only the uncertainty interval of the full cell is plotted. As can be observed, the trend for the full cell is well bounded for long‐term predictions, but it may not capture the variations in the measurement data. In Figure 7, the trends for the long‐term predictions of the half‐cell, electrode, and interfacial contact are also plotted and compared with the prediction for the full‐cell voltage. A degradation of 5% and 10% in the full‐cell voltage can also be predicted from the plot, corresponding to 1613 and 2122 hours of operation time, respectively. To validate the effectiveness of the model, we performed durability measurements on a standard cell without interfacial sensors under identical operating conditions. The regular cell exhibited a voltage of 1.33 V at a current density of 1.86Acm^−2^, which is comparable to the performance of the cell with interfacial sensors. The results, depicted in Figure S4 (Supporting Information), indicate that the regular cell without sensors reached a 5% degradation threshold after 1627 h, exhibiting only a 0.8% deviation from the predicted value (1613 h in Figure 7). Both the hydrogen electrode/electrolyte half‐cell and the PNC electrode/electrolyte interface remain stable for up to 2922 h. However, both the PNC electrode and the electrode/electrolyte interface contribute to the overall degradation of the electrolysis cell. The root cause of the electrolysis performance degradation has been identified as an increase in voltage induced by the PNC bulk electrode, even though its absolute value is smaller than that of the interfacial contact, even before the cell reaches 10% performance degradation. These synergistic findings suggest that the anode‐supported half‐cell with an acid‐treated electrolyte surface exhibits excellent physicochemical durability under steam electrolysis at 600 °C. Nevertheless, there is still room for improvement in the long‐term stability of the PNC cathode, despite its exceptional initial performance. Future research will focus on utilizing the model developed as a diagnostic tool to detect anomalies, classify fault modes, and combine with feature extraction methods to identify the causes of failure in the large scaled PCECS and PCEC stacks. Future studies can also benefit from increased sample sizes to further enhance statistical reliability.

## 3 Conclusion

Data‐driven modeling shows promise for diagnosing and predicting the performance of intermediate‐temperature ceramic electrochemical cells, enabling their development, manufacturing, and optimization. In this study, we employed machine learning to analyze electrochemical results and optimize simulation models for failure analysis and lifetime prediction, using real‐time electrochemical data from in‐house fabricated PCECs with embedded Pt electric interfacial sensors operating under steam electrolysis conditions at 600 °C. The voltage corresponding to full‐cell, half‐cell, PNC electrode, and PNC/electrolyte interfacial contact were quantitively monitored for 1171 h and the trends were predicted up to 2922 h. The results showed that the anode‐supported half‐cell with an acid‐treated electrolyte surface exhibits excellent physicochemical durability under steam electrolysis at 600 °C and the root cause of the electrolysis performance degradation has been identified as an increase in voltage induced by the PNC bulk electrode. Our approach can complement physical and semi‐empirical models, as well as specialized diagnostics, and highlights the promise of combining data generation and data‐driven machine learning for understanding and developing complex systems such as PCECs.

## 4 Experimental Section

### Fabrication of PCEC with Interfacial Electrical Sensor

In this study, a NiO‐BaZr_0.4_Ce_0.4_Y_0.1_Yb_0.1_O_3_ (BZCYYb4411) electrode‐supported cell with a diameter of Ø 1.0″ was used. The BZCYYb4411 was prepared in‐house through solid‐state reaction, as described in previous work^[^ ^22^ ^]^ with a configuration of a NiO‐BZCYYb (500 µm‐thick) anode support, a BZCYYb (12 µm‐thick) electrolyte, and a PrNi_0.5_Co_0.5_O_3−δ_ (PNC) (30 µm‐thick) cathode. During fabrication, Pt electric sensors were printed at the interface between the electrode and electrolyte. The electrode support half‐cell was first fabricated using a well‐established tape casting and firing method with 10‐minute acid etch treatment.^[^ ^2b^ ^]^ Afterward, a Ø 0.5″ Pt ring was printed on the electrolyte surface with thickness of 0.03″(0.76 mm), followed by drying at 80 °C for 30 min. Then, a 30 µm‐thick PNC cathode was screen printed onto the electrolyte to cover the Pt ring and annealed together at 1030 °C for 2 h to form the designed cell.

### Electrochemical Testing and Data Generation

To function as the current collecting layer for the steam and hydrogen electrodes, a Pt paste (Fuel Cell Material) was applied on both sides of the cell. The cell was sealed using Aremco cerambond 552 sealant and maintained at 600 °C for 2 h. Steam electrolysis was performed after reducing the hydrogen electrode in a pure H_2_ atmosphere of 60 ml min^−1^ for 2 h. During operation, the gas composition was 60 ml min^−1^ of H_2_ on the hydrogen electrode side and 30% steam + 70% oxygen (120 ml min^−1^) on the steam electrode side. The current–voltage characteristic curves were collected using a Solartron 1400 and 1470 electrochemical working station. During long‐term operation, constant current density of −1.8 Acm^−2^ was applied which corresponds to an electrolysis voltage of 1.31 V, close to the thermal neural voltage of steam electrolysis at 600 °C. Additionally, the electrochemical impedance spectra (EIS) of each cell component were collected at different periods. The frequency range of EIS measurement was between 0.1 and 1 × 10^5^ Hz with a 10 mV amplitude. The ohmic resistance and the polarization resistance of the cell were derived from the EIS. Figure S5 (Supporting Information) illustrates the electric leads setup for the electrochemical tests. For *V–I* test, the voltage variation across the half‐cell, which includes the anode and electrolyte, was denoted as V_h_ (measured by V_3‐4_). The overall applied electrolysis potential was represented as V_f_ (measured by leads V_1‐4_) while the potential generated by the PNC oxygen electrode was denoted as V_o_ (measured by V_1‐3_). Figure S5b,c (Supporting Information) show the electric leads setup for EIS measurement of full‐cell and half‐cell respectively. The post‐test samples were examined using a scanning electron microscope (SEM, FEI Quanta 250 FEG) to observe the cross‐sectional variation. For element mapping in the transmission electron microscope (JEOL JEM‐2010 FASTEM), the sample was prepared using the focused ion beam technique (Thermo Fisher, Strata 400).

### Machine‐Learning Model Development and Training

To enhance the deployment and life management of PCEC systems, machine learning techniques can serve as prognostic and diagnostic tools to better comprehend the aging phenomena and emulate the behavior of the whole PCEC systems. As illustrated in Figure 7, two types of machine learning techniques, namely modular regression model and deep neural network^10^, were employed to predict PCEC systems’ remaining useful life (RUL) and assess the health state, respectively. Furthermore, outlier detection techniques like isolation forest or robust covariance were used to eliminate spikes and dips in the measured data, and locally weighted scatter smoothing was implemented to smooth and clean the data. Many machine learning applications required the ability to distinguish whether a new data point belongs to the same distribution as existing historical data, i.e., an inlier, or should be regarded as an anomaly, i.e., an outlier. By means of outlier detection, machine learning models attempt to fit the areas where the training data is most concentrated, while ignoring the deviant/anomalous data points. Smoothing is another technique used to eliminate noise from the measured data, which can aid in identifying trends in the data, particularly for long‐term degradation prediction, which is one of the main objectives.

More experimental and computational details can be found in the Appendix S1 (Supporting Information).

## Conflict of Interest

The authors declare no conflict of interest.

## Supporting information

Supporting Information

## Data Availability

The data that support the findings of this study are available in the supplementary material of this article.
