# Supplementary material for: Root Cause Analysis of Degradation in Protonic Ceramic Electrochemical Cell with Interfacial Electrical Sensors Using Data‐Driven Machine Learning
Source: Adv Sci (Weinh). 2023 Aug 26;10(30):2304074. doi: 10.1002/advs.202304074 (PMC10602546; doi:10.1002/advs.202304074)
Supplement: Supplementary file 1 — Supporting Information [file ADVS-10-2304074-s001.pdf]

ADVANCED SCIENCE Open Access

Supporting Information

for *Adv. Sci.*, DOI 10.1002/advs.202304074

Root Cause Analysis of Degradation in Protonic Ceramic Electrochemical Cell with Interfacial Electrical Sensors Using Data-Driven Machine Learning

Wei Wu, Congjian Wang*, Wenjuan Bian, Bin Hua, Joshua Y. Gomez, Christopher J. Orme, Wei Tang, Frederick F. Stewart and Dong Ding*

Supporting Information

Root Cause Analysis of Degradation in Protonic Ceramic Electrochemical Cell with Interfacial Electrical Sensors using Data-driven Machine Learning

Wei Wu*, Congjian Wang*, Wenjuan Bian, Bin Hua, Joshua Y. Gomez, Christopher J. Orme, Wei Tang, Frederick F. Stewart and Dong Ding,

Wei Wu, Wenjuan Bian, Bin Hua, Dong Ding, Joshua Y. Gomez, Christopher J. Orme, Wei Tang, and Frederick F. Stewart

Energy & Environmental Science and Technology, Idaho National Laboratory, Idaho Falls, Idaho 83415, USA

Congjian Wang

Nuclear Science and Technology, Idaho National Laboratory, Idaho Falls, Idaho 83415, USA

E-mail: wei.wu@inl.gov, congjian.wang@inl.gov

Experimental Section/Methods

Machine-learning model development and training

Outliers detection: The technique of robust covariance assumes that normal operation data is drawn from a known distribution, such as a Gaussian distribution[1]. Using this assumption, elliptic envelope is utilized to define the "shape" of regular data and label the points outside the envelope as outliers. An alternative method is to use an isolation forest to detect anomalies[2]. The isolation forest isolates observations by randomly selecting a feature and a split value between the maximum and minimum values of the selected feature. The path length from the root node to the terminating node is used as a measure for anomalies. In this work, both the robust covariance and isolation forest techniques were used to detect the outliers of degradation data. The measured full cell voltage data was used as input, and the Scikit-Learn tool was employed (both methods are available in the current version of Scikit-Learn) to identify the outliers. The outliers fraction was set to 10%. Figure. S6 and Figure. S7 demonstrates the detected outliers (blue dots) versus the regular data (orange dots). As illustrated in these two figures, both methods successfully identify large unpredictable spikes or dips, which can have negative impacts during the prediction of the degradation process.

Locally weighted scatterplot smoothing (LOWESS): The manuscript employs locally weighted scatterplot smoothing (LOWESS) to smooth the measured data. LOWESS is a scatterplot smoothing method that has been implemented in statsmodel[3]. Suppose that the input data contains n points, denoted by (x_i, y_i), where $i = 1, \cdots, n$), the algorithm estimates the smooth valuey_k by considering some closest points to (x_k, y_k), and using a weighted linear regression to fit their values, in which the fitted at x_k is the estimated y_k. The weight assigned to (x_j, y_j) is the tricube function applied to $abs(x_k - x_j)$.

Modular regression model: The modular regression model, the Prophet model, used in this work was proposed and developed by Facebook to perform business forecasts at scale[4]. It attempts to resolve several issues in existing forecasting models, including changes in trend, outliers, multiple strong seasonalities, and regular operations/events effects. We provide a brief illustration of the Prophet model and offer some customized explanations for the PCEC prognostic analysis in this manuscript.

In Prophet forecasting model, the time series are decomposed into three main components:

$$V(t) = g(t) + s(t) + o(t) + \epsilon_t \quad (1)$$

Where $g(t)$ is the trend function which models non-periodic changes in the value of the degradation data, $s(t)$ represents periodic changes (e.g., weekly/monthly/yearly seasonality), and $o(t)$ represents the effects of dynamic operations which occur on regular schedules. The white noise term ϵ_t, assumed normally distributed, represents the changes which are not accommodated by the model.

Prophet model utilizes a classic approach to model the trend, i.e., a combination of an offset m and a growth rate k. The trend effect at a time t_i is given by multiplying the growth rate k_i by the difference in time:

$$g(t_i) = m_i + k_i \Delta t_i = g(t_{i-1}) + k_i\,(t_i - t_{i-1}) \quad (2)$$

The Prophet model allows for the growth rate to vary at multiple locations, resulting in a trend modelled as a continuous piece-wise linear function. This produces an interpretable, yet non-linear form of the trend. In other words, the trend effect within a time window delimited by two change points is determined by the steady growth rate multiplied by the time difference. The Prophet model incorporates changes in the trend by explicitly defining changepoints where the growth rate is allowed to change. Suppose there are S changepoints at times s_j, $j = 1, \cdots, S$. A vector of rate adjustments $\delta \in R^S$, where δ_j is the change in rate that occurs at time s_j. The rate at any time t_I is

then the base rate k_0, plus all the adjustments up to that point: $k_0 + \sum_{j=1}^{I} \delta_j$. To avoid overfitting on a small number of final points, the final trend segment is set to a larger set of observations (20% of training data by default). To make predictions into the future, the Prophet model uses the final growth rate to linearly extrapolate the trend.

Fourier series is used to model the periodic effects. Let p be the regular period, and assume the data are scaled in days, the periodic changes can be approximated with as standard Fourier series.

$$s(t) = \sum_{n=1}^{N} \left(a_n \cos\left(\frac{2\pi nt}{p}\right) + b_n \sin\left(\frac{2\pi nt}{p}\right) \right) \quad (3)$$

In addition, regular operations/events such as characterization or dynamic operations can cause unpredictable spikes or dips, which can negatively impact the prediction of the degradation process. Since these events often do not follow a periodic pattern, their effects cannot be accurately modeled by a smooth cycle. The Prophet model allows the analyst to provide a customized list of past and future operations/events. If the impact of these events on the degradation data is similar, Prophet uses a matrix of regressors on the regular events to incorporate their effects on long-term predictions.

Prophet model assumes the future trend changes will have the same average frequency and magnitude of rate changes as it has in history. It first specifies many potential changepoints at which the rate is allowed to change followed by Laplace distribution with mean zero and prior scale parameter λ. Bayesian inference or maximum likelihood estimated is utilized to estimate the posterior of these scale parameters that will be employed to simulate future rate changes. In this case, the uncertainty in the forecast trend can be computed using simulated future rate changes. Thus, the prior scale parameter plays a critical role in the determination of trend changes, i.e., increasing it will make the trend more flexible (tend to overfit) while decreasing it will make the trend less flexible (tend to underfit). When visualizing the future predictions, this parameter can be adjusted as needed if the trend seems to be over- or under-fit. This parameter can also be automatically determined via a cross validation approach. In this research, we manually tested the prior scale parameters to find the best values for these parameters.

Deep neural network model: As illustrated in many literatures, deep learning methods like Long short-term memory (LSTM) can be used to predict time series10. The simple neural network is known as a feedforward neural network, which consists of three layers: an input layer, a hidden layer, and an output layer. In this architecture, there are no direct connections or feedback loops between neurons in the hidden layer. To enhance prediction accuracy over time, a recurrent neural

network (RNN) is introduced, which leverages recurrent connections between neurons in the hidden layer to combine current inputs with inputs that the neurons have previously perceived in time. To further improve the performance and avoid the gradient exploding and vanishing problem, LSTM adds an input gate, a forget gate and an output gate to RNN network. The input gate quantifies the importance of new information carried by the input, the forget gate allows the LSTM cell to remember or forget the state from the previous step, and the output gate filters the output data to determine whether it should influence other neurons. This design of LSTM architecture helps in improving prediction accuracy and mitigating potential issues during training and inference.

In this study, we will omit the illustration of the LSTM network and instead focus solely on its application to PCEC degradation forecasting. This decision is based on the fact that LSTM has been extensively employed in various scientific and engineering fields, and information on its architecture can be easily found online. To facilitate a better understanding of the analysis results, we will provide a brief introduction to the concept of data windows for LSTM. The LSTM model generates predictions based on a data window, which comprises consecutive samples from the input data. The main features of the windows are:

1. The width (number of time steps) of input and output windows

2. The time offset (i.e., shift) between them

As an example, when predicting 24 hours into the future based on 24 hours of historical data, the data window is depicted in Figure. S8a. Conversely, a model that predicts one hour into the future with the same 24 hours of historical data would require a different window, illustrated in Figure. S8b. The input time series is partitioned using this data window to generate batches of time series inputs and targets (i.e., outputs/forecasts), which are then used to train the LSTM model. Paste your materials and methods section here.

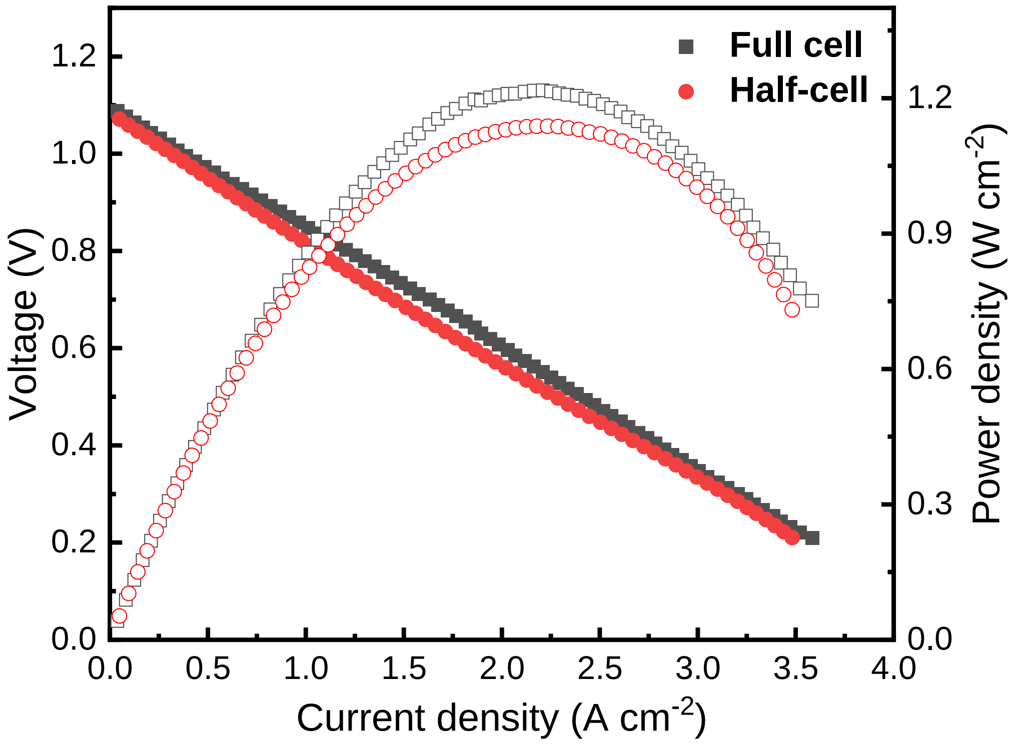

Figure S1. I-V and I-P curves of full cell and half-cell at 600 oC.

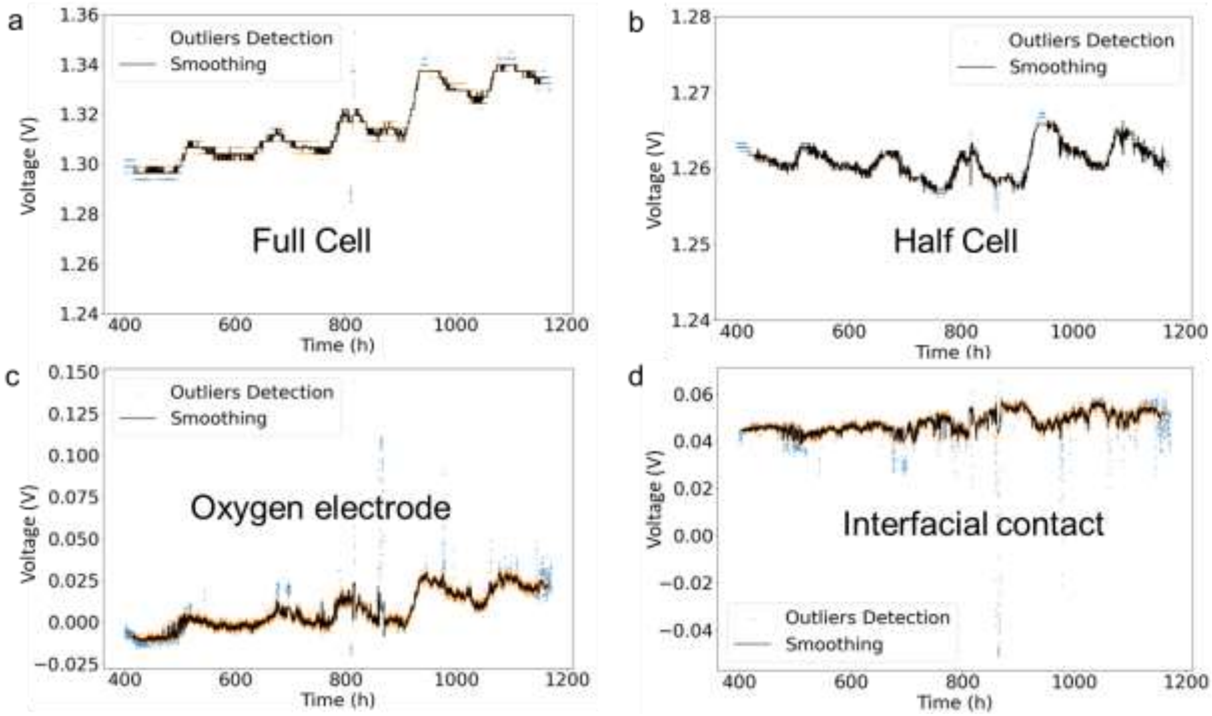

Figure S2. Outliers’ detection and data smoothing for raw data.

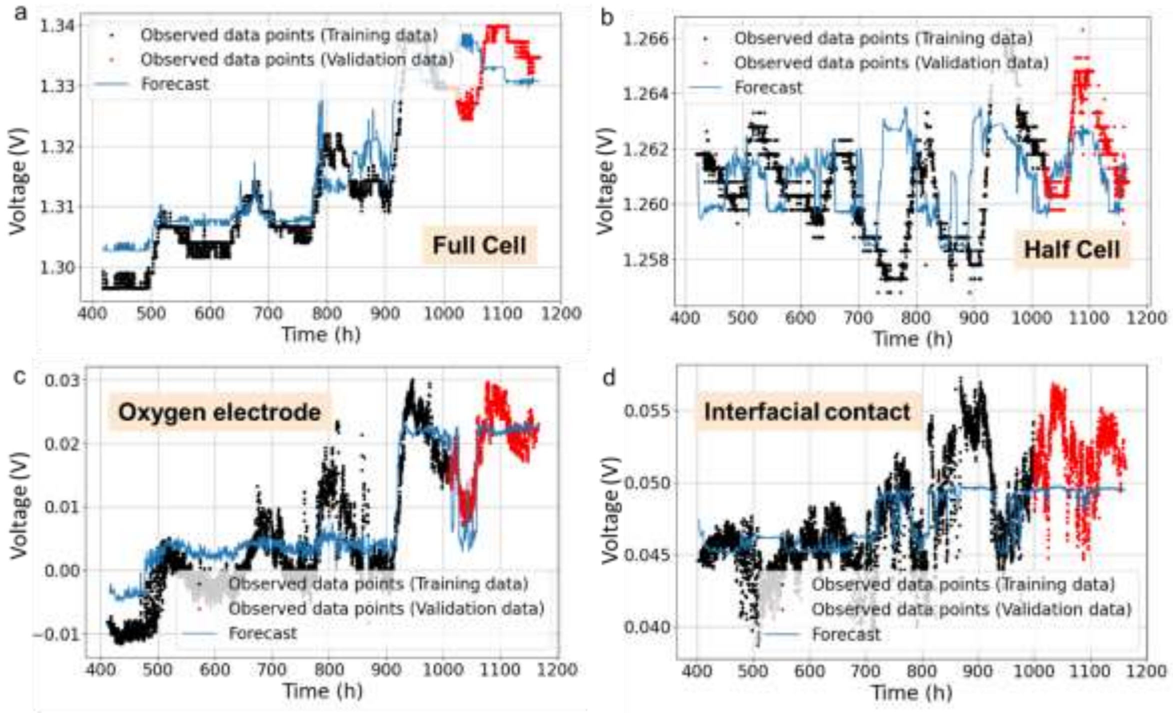

Figure S3. Voltage predictions for next 1 day (24 hours) using LSTM.

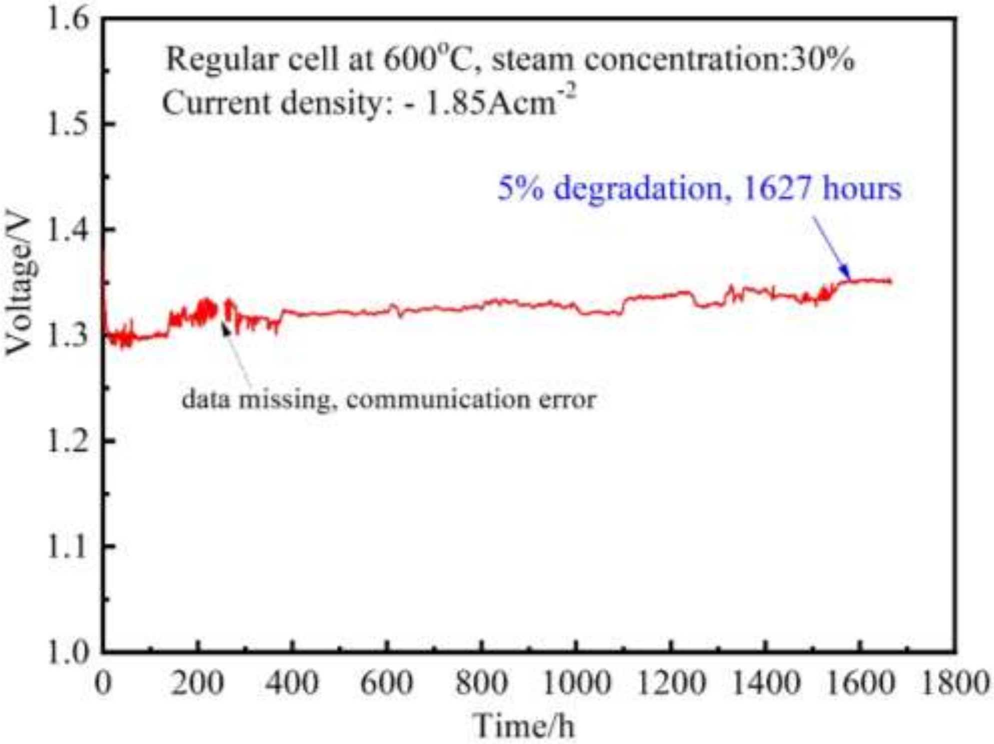

Figure S4. Longterm durability of regular cell without interfacial sensors.

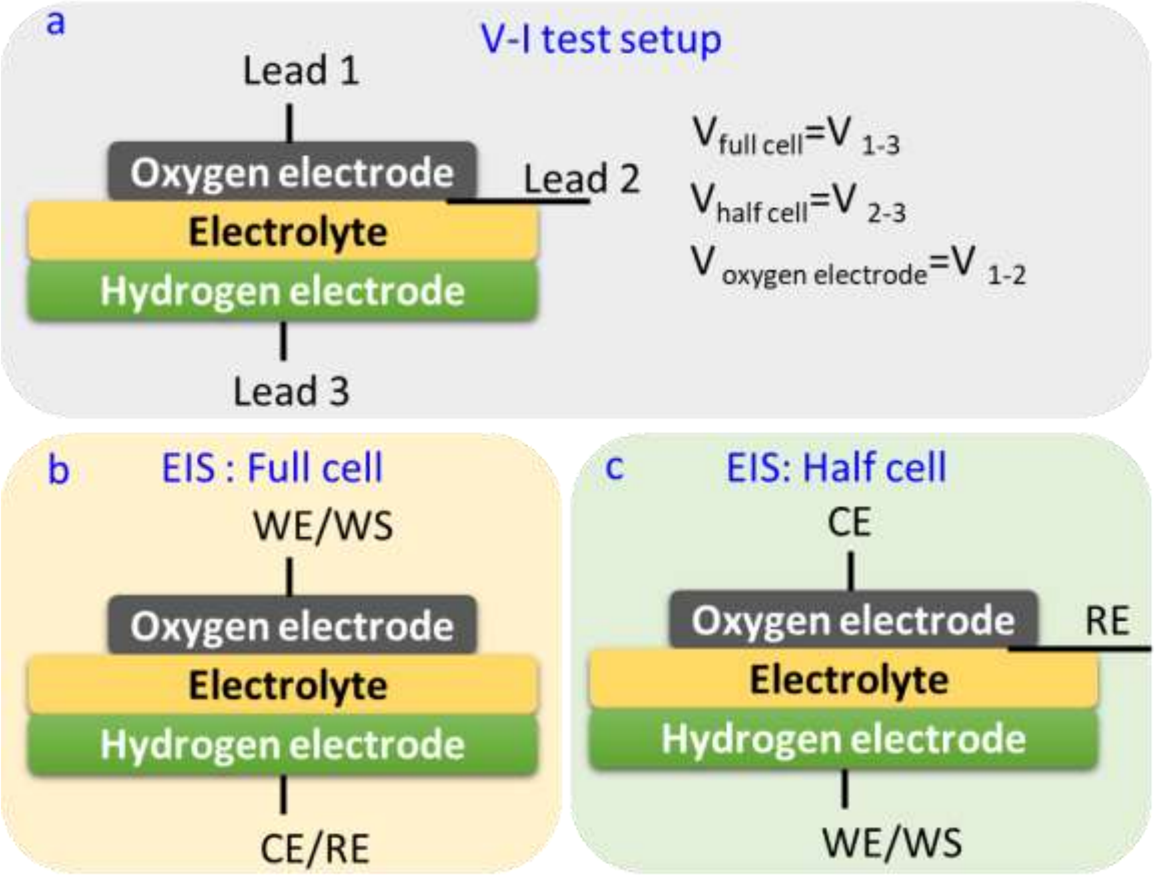

Figure S5. Electrical leads setup for a) V-I test and (b, c) EIS measurements.

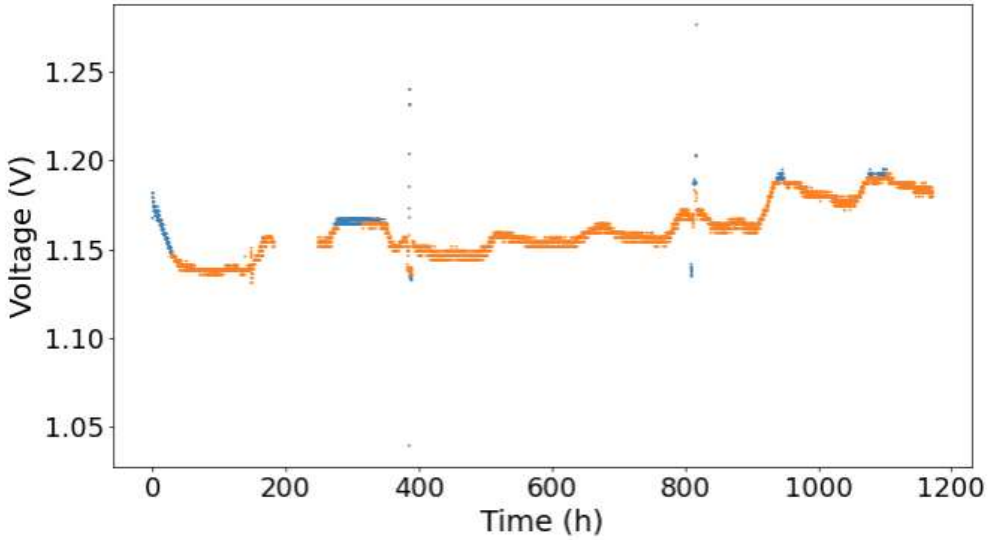

Figure S6. Outliers detected by robust covariance with outliers fraction = 0.1, outliers (blue dots) vs. inliers (orange dots).

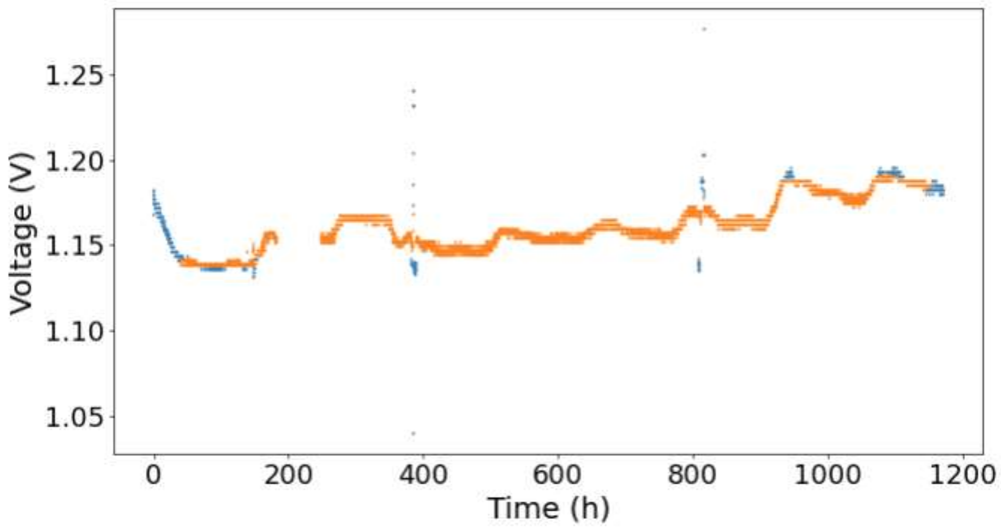

Figure S7. Outliers detected by isolation forest outliers fraction = 0.1, outliers (blue dots) vs. inliers (orange dots).

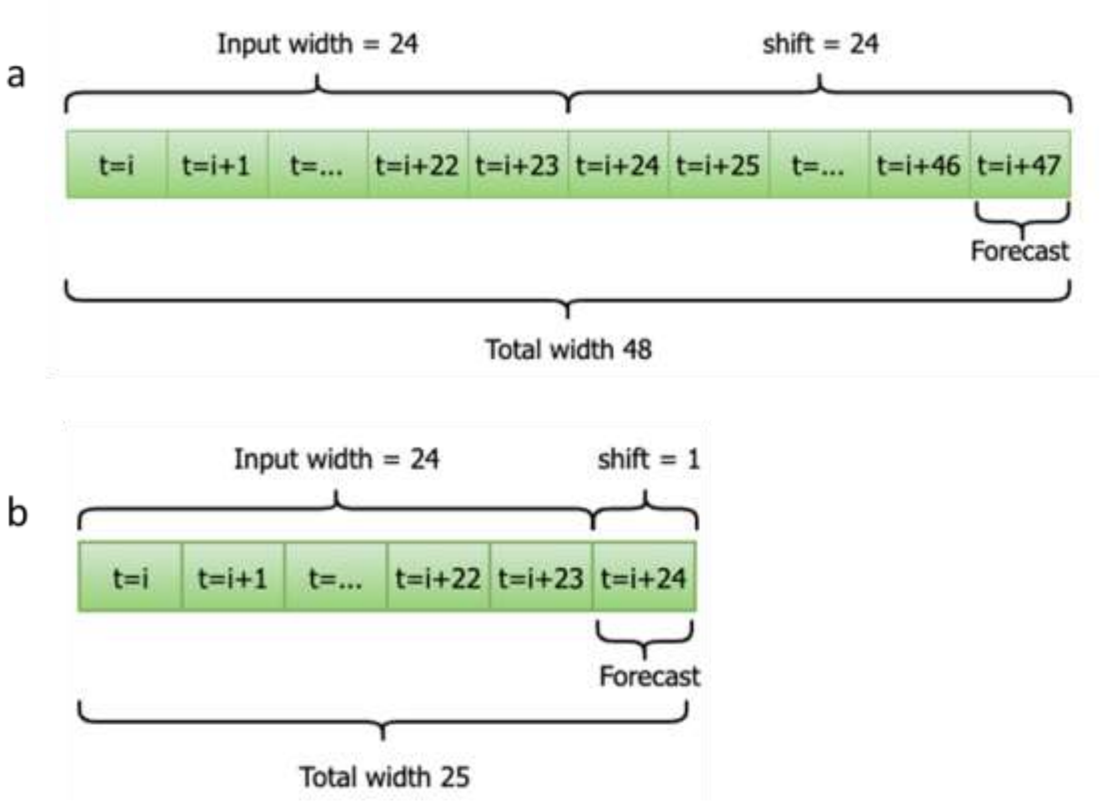

Figure S8. Data window for (a) long and (b) short period prediction.

Table S1. LSTM structure.

Layer (type)	Output Shape	Param #
LSTM	(1, None, 32)	4352
Dense	(1, None, 1)	33
Lambda	(1, None, 1)	0
Total params: 4,385; Trainable params: 4,385; Non-trainable params: 0		

Table S2. Prediction errors for validation data using LSTM.

Error Metrics	Full Cell		Half Cell		Electrode		Interfacial	
	1 hour	1 day	1 hour	1 day	1 hour	1 day	1 hour	1 day
MSE	4.91E-6	5.22E-5	1.89E-7	2E-6	7.92E-6	1.99E-5	1.19E-6	1.1E-5
MAPE	1.53E-3	5.04E-3	2.88E-4	1E-3	2.36E-1	2.26E-1	1.62E-2	5.33E-2
R2	0.788	-1.26	0.929	0.248	0.751	0.376	0.8	-0.858
MAE	2.04E-3	6.72E-3	3.64E-4	1.27E-3	1.95E-3	3.65E-3	8.38E-4	2.83E-3

Table S3. Prediction errors from Modular regression models

Error Metrics	Full Cell	Half Cell	Electrode	Interfacial
MSE	2.5E-06	2.96E-7	5.68E-6	3.53E-6
MAPE	9.3E-4	3.37E-4	2.32	3.0E-2
R2	0.986	0.918	0.952	0.757
MAE	1.22E-3	4.25E-4	1.76E-3	1.43E-3

Reference

[1] P. J. Rousseeuw, K. V. Driessen, Technometrics 1999, 41, 212.

[2] F. T. Liu, K. M. Ting, Z.-H. Zhou, presented at 2008 eighth ieee international conference on data mining 2008.

[3] W. S. Cleveland, Journal of the American statistical association 1979, 74, 829.

[4] S. J. Taylor, B. Letham, The American Statistician 2018, 72, 37.
